# Replacement of the Mouse LD_50_ Assay for Determination of the Potency of AbobotulinumtoxinA with a Cell-Based Method in Both Powder and Liquid Formulations

**DOI:** 10.3390/toxins15050314

**Published:** 2023-04-29

**Authors:** Elena Fonfria, Elizabeth Marks, Lisa-Marie Foulkes, Rebecca Schofield, Daniel Higazi, Sam Coward, Alistair Kippen

**Affiliations:** 1 Ipsen Bioinnovation, Abingdon OX14 4RY, UK; 2 Ipsen Biopharm Ltd., Wrexham LL13 9UF, UKrebecca.schofield@ipsen.com (R.S.); daniel.higazi@ipsen.com (D.H.); alistair.kippen@ipsen.com (A.K.)

**Keywords:** botulinum, toxin, LD_50_, CBA, potency, replacement

## Abstract

Botulinum neurotoxins (BoNTs) are important therapeutic agents. The in vivo median lethal dose (LD_50_) assay has been commonly used to measure the potency of BoNT commercial preparations. As an alternative, we developed cell-based assays for abobotulinumtoxinA in both powder (Dysport^®^, Azzalure^®^) and liquid (Alluzience^®^) formulations using the in vitro BoCell^®^ system. The assays demonstrated linearity over 50–130% of the expected relative potency, with a correlation coefficient of 0.98. Mean recoveries of 90–108% of the stated potency were observed over this range. The coefficients of variation for powder and liquid formulations, respectively, were 3.6% and 4.0% for repeatability and 8.3% and 5.0% for intermediate precision. A statistically powered comparability assessment of the BoCell^®^ and LD_50_ assays was performed. Equivalence was demonstrated between the assays for the liquid formulation at release and end of shelf life using a paired equivalence test with predefined equivalence margins. For the powder formulation, the assays were also shown to be equivalent for release samples and when determining loss of potency following thermal degradation. The BoCell^®^ assay was approved for establishing the potency of abobotulinumtoxinA for both powder and liquid formulations in Europe and for the powder formulation only in the USA.

## 1. Introduction

Botulinum neurotoxins (BoNTs) are important therapeutics used widely in many medical applications, such as the treatment of neuromuscular spasms (e.g., spasticity and cervical dystonia). The therapeutic utility of BoNTs is expanding, and novel indications continue to emerge [1,2]. BoNTs are also widely used in aesthetic medicine.

All BoNTs are modular proteins consisting of a heavy chain and a light chain linked by a disulfide bond [3,4]. The activity of BoNTs involves a multistep process of heavy-chain-mediated cell binding to neuronal cells, endocytosis, and translocation of the light chain into the cytosol, followed by cleavage by the light chain of soluble N-ethylmaleimide-sensitive factor attachment receptor (SNARE) proteins [3,4]. Cleavage of SNARE proteins by the zinc endopeptidase action of the light chain results in disrupted fusion of synaptic vesicles containing neurotransmitters with the plasma membrane; this blocks the release of neurotransmitters and paralyzes muscle activity [3,4]. BoNT serotypes differ in their target SNARE proteins and cleavage sites; for example, BoNT-A and -E cleave synaptosomal-associated proteins of 25 kDa (SNAP-25), whereas BoNT-B, -D, -F, and -G cleave vesicle-associated membrane proteins, and BoNT-C cleaves both SNAP-25 and syntaxin [3,4].

There are several commercially available BoNT-A-containing products that are not medically interchangeable owing to differences in manufacturing processes and their measurement of potency; as such, each BoNT product has its own indications and dosage [5,6,7]. Historically, commercial BoNT-A products have been formulated as a powder for reconstitution prior to injection. However, new products in a ready-to-use liquid formulation are coming to market, with products being approved in Korea [8] and in Europe (abobotulinumtoxinA (aboBoNT-A) in a novel liquid formulation (Alluzience^®^)). Potency testing is a requirement for the registration of pharmaceuticals for human use [9]. The mouse median lethal dose (LD_50_) assay has long been used to assess BoNT potency. However, several limitations are associated with the LD_50_ assay, including extensive animal use, requirements for highly skilled personnel and facilities, lack of automation, and laboratory-dependent outcomes [9,10]. These drawbacks have led to the search for alternative and reliable approaches to assess the potency of BoNTs, with the consideration that any replacement potency assay must reflect all steps in the cellular intoxication pathway of BoNTs [10,11,12,13]. Although several alternative methodologies have been described previously, these have often been limited to the detection of the BoNT protein or have only assessed a single element of the BoNT mechanism of action, such as endopeptidase activity. Frequently proposed alternatives to the LD_50_ have been published without sufficient consideration of the regulatory and commercial requirements to be able to adopt these methodologies for the testing of pharmaceutical products [14]. The progress in the development of in vitro cell-based methods for BoNT potency determination provides an approach that incorporates the key aspects of the BoNT mechanism of action.

**Figure 1:**
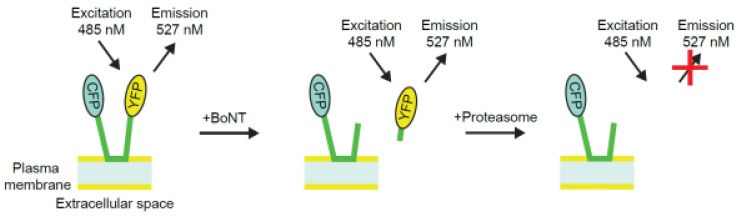
Cartoon representation of the BoCell^®^ cell-based assay (adapted from [18]). BoNT, botulinum neurotoxin; CFP, cyan fluorescent protein; YFP, yellow fluorescent protein.

Dong et al. developed fluorescence reporters that respond to BoNT activity, including activity in living cells [15]. The reporter technology was further developed by BioSentinel Inc. (Madison, WI, USA) and used to develop the novel BoCell^®^ cell line and assay [16,17], which was subsequently licensed by BioSentinel Inc. to Ipsen Biopharm Ltd (Wrexham, UK). The BoCell^®^ cell line is an engineered murine neuroblastoma cell line that stably expresses a reporter composed of SNAP-25 fused to cyan and yellow fluorescent proteins (CFP, YFP) on the N- and C-terminals of SNAP-25, respectively. Following exposure, BoNT-A binds to and is then internalized by the cells where the light chain cleaves the SNAP-25 moiety of the reporter. Following cleavage, the C-terminal fragment containing the YFP fluorophore is rapidly degraded. Loss of YFP fluorescence relative to the BoNT-insensitive CFP fluorescence provides the quantitative readout of the assay (Figure 1) [16,17].

The objective of the current study was to assess the suitability of a potency method that uses the BoCell^®^ assay as a replacement for the LD_50_ assay to test aboBoNT-A in both powder (Dysport^®^, Azzalure^®^) and liquid (Alluzience^®^) formulations. Specifically, the goal was to validate the BoCell^®^ assay to determine its capability as a release test for commercial batches and to conduct a statistically powered comparability assessment of data from the cell-based assay (CBA) and the LD_50_ assay using release and stability samples of aboBoNT-A in both powder and ready-to-use liquid formulations.

## 2. Results

### 2.1. Assay Specificity and Demonstration of Toxin Uptake

AboBoNT-A (Ipsen, Paris, France) at a vial strength of 500 LD_50_ units (U) was used, with further additional confirmatory analyses being conducted with 125 U/vial for some evaluations, as indicated.

**Figure 2:**
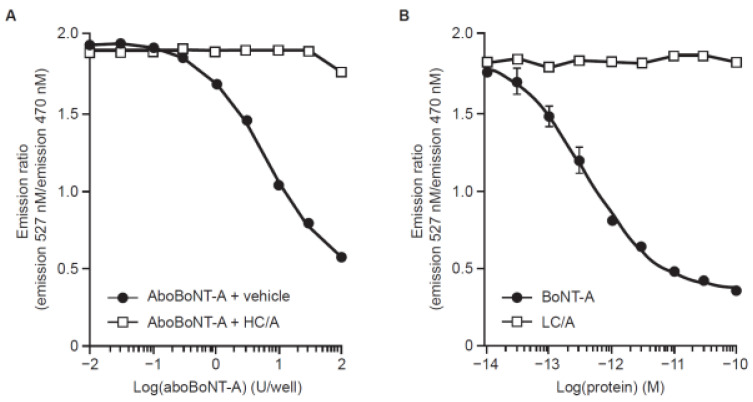
Concentration–response curves for demonstration of toxin-binding requirement: (**A**) aboBoNT-A, or aboBoNT-A together with 5 pM HC/A; and (**B**) BoNT-A or LC/A, lacking receptor-binding activity. AboBoNT-A, abobotulinumtoxinA; BoNT-A, botulinum toxin A; HC/A, heavy-chain receptor-binding domain of BoNT-A; LC/A, light chain of BoNT-A; U, units.

When the BoCell^®^ cell line was incubated with a range of dilutions of aboBoNT-A or BoNT-A, a concentration–response relationship was observed (Figure 2). A control sample that contained only excipients (i.e., without aboBoNT-A, placebo) showed no response with the BoCell^®^ assay; thus, specificity (i.e., the sensitivity of the fluorescence reporter in the BoCell^®^ cells to aboBoNT-A activity) was established. Assay responsiveness was reduced when BoCell^®^ cells were incubated with 5 pM of a fragment of BoNT-A that contained the heavy-chain receptor-binding domain of BoNT-A (HC/A), together with increasing concentrations of aboBoNT-A (Figure 2A). The receptor-binding domain acts as a competitive antagonist of the full-length toxin; therefore, inhibition of assay responsiveness indicates that the effect of aboBoNT-A requires its binding domain (widely accepted as the first step in the cellular mode of action of BoNTs). In addition, the BoCell^®^ assay was not responsive to a recombinant preparation that contained the light chain of BoNT-A (LC/A), which lacked receptor-binding activity but had the proteolytic activity of the light chain (Figure 2B). Again, this indicates that BoNT-A cellular receptor-binding activity is required to elicit a response in the BoCell^®^ assay. These data showed that the developed cell line exhibited selective concentration responsiveness and that it reflected key steps of the cellular intoxication pathway.

### 2.2. Assay Linearity, Accuracy, Repeatability, and Intermediate Precision of the BoCell^®^ Assay

**Figure 3:**
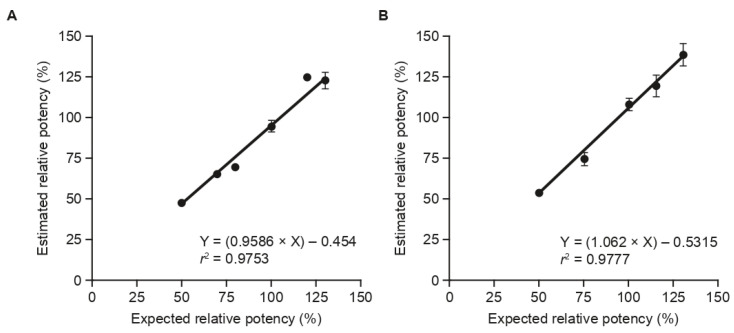
Linearity of the BoCell^®^ assay for detecting the potency of aboBoNT-A in: (**A**) powder formulation at 500 U/vial; and (**B**) liquid formulation at 200 U/vial. AboBoNT-A, abobotulinumtoxinA; U, units.

Following the successful development of the assay for testing aboBoNT-A in both powder and liquid formulations, we progressed to validation of the assay. The potency method for aboBoNT-A was a relative potency method, with a test and reference standard on each plate. To determine the potency for the powder formulation, a nonlinear quantitative response analysis using a four-parameter logistic curve fit was utilized; for the liquid formulation, a linear model utilizing parallel line analysis was employed. The BoCell^®^ assay was validated according to requirements from the International Conference of Harmonisation [19] and guidance from the US Food and Drug Administration (FDA) [20]. The predefined acceptance criteria were selected for this assay based on the requirements of the method’s intended purpose (i.e., for lot release and stability potency testing of aboBoNT-A) and were in line with widely used criteria in the field of CBA development for biologics. A summary of the predefined criteria is presented in Table 1. Using an aboBoNT-A powder formulation at 500 U/vial and a liquid formulation at 200 U/vial, the linearity of the analytical procedure was tested at 50%, 70%, 80%, 100%, 120%, and 130% (for powder) and 50%, 75%, 100%, 115%, and 130% (for liquid) of the expected relative potency to assess whether results obtained with the BoCell^®^ assay were directly proportional to the concentration of aboBoNT-A in the sample. When estimated potencies were plotted against the expected results, linearity was observed, with correlation coefficients (*r*^2^) of 0.9753 and 0.9777 for the powder and liquid formulations, respectively (Figure 3, Table 1). When the sample concentration was tested at a nominal 130%, relative to the reference standard in five and six independent assays for the powder and liquid formulations, respectively, accuracy was 94%, 95%, 94%, 101%, and 90% for powder and 102%, 112%, 102%, 108%, 108%, and 112% for liquid. At the nominal 100% level, accuracy was 91%, 98%, 96%, 99%, 95%, and 91% for powder and 108%, 114%, 105%, 109%, 105%, and 105% for liquid and, at the nominal 50% level, accuracy was 93%, 103%, and 96% for powder and 102%, 110%, 111%, 104%, 112%, and 99% for liquid. All results met the predefined acceptance criteria. Overall, recovery of 90–103% for powder and 99–112% for liquid of the stated potency over the range of 50–130% was recorded, which is within the acceptance criteria for assay accuracy (Table 1).

The repeatability of the assay for the powder formulation was assessed using 500 U/vial and 125 U/vial samples of aboBoNT-A. When each sample was assayed six times, the coefficient of variation (%CV) was 3.6% for the 500 U/vial sample and 3.3% for the 125 U/vial sample (Table 1 and Table 2). To assess the repeatability of the assay with the liquid formulation, the %CV from results obtained for the same sample, assessed in three assays and performed by the same analyst at three test levels (total of nine results), was calculated, and a %CV of 4% was obtained. The results for both powder and liquid formulations were within the acceptance criterion of a %CV of 15% or below for repeatability.

When the powder formulation was tested by different analysts on different days to evaluate intermediate precision, a difference of 1.6% was observed between the means of the two analysts’ data for 500 U/vial samples, with an overall %CV of 8.3%, demonstrating acceptable intermediate precision (Table 1 and Table 3). For the liquid formulation, the %CV of the six reportable results at each test level was calculated. For intermediate precision to be considered acceptable, each level must have a %CV of 15% or below. The results are summarized in Table 4.

The performance of the BoCell^®^ assay for aboBoNT-A was similar to the mouse LD_50_ assay when tested using 500 U/vial samples. The *r*^2^ correlation coefficient for the linearity of the mouse LD_50_ assay over a range of 50–130% was 0.99, and accuracy was 99–104%. For repeatability, triplicate determinations of potency were performed with a 500 U/vial sample diluted to 50%, 100%, and 130% of the target, and the %CV ranged from 4% to 10% over the three dilutional levels tested. For intermediate precision, six assays at the 100% level were performed, and the %CV of the LD_50_ assay was 2% with a 500 U/vial sample.

### 2.3. Comparability Assessment of Data from the LD_50_ Assay and BoCell^®^ Assay Using Release and Stability Samples

Release samples from 30 batches of aboBoNT-A in the powder formulation and 11 batches of aboBoNT-A in the liquid formulation were used in the comparability studies. The mean (range) result from the BoCell^®^ assay, as a percentage of the LD_50_ assay result, was 102% (89–112%) for powder and 101% (83–122%) for liquid.

**Figure 4:**
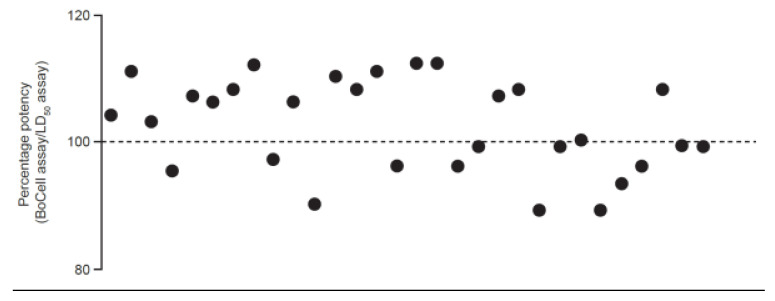
Graphical representation of paired potency results from the mouse LD_50_ assay and BoCell^®^ assay using release samples of aboBoNT-A at 500 U/vial. AboBoNT-A, abobotulinumtoxinA; LD_50_, median lethal dose; U, units.

Figure 4 shows a graphical representation of paired potency results from the LD_50_ assay and BoCell^®^ assay for samples of 500 U/vial powder product, and Table 5 shows the summarized potency data for the liquid formulation batches. Results for the comparison of variance between the LD_50_ and CBA release comparability data sets indicated significantly higher variability in potency results from the BoCell^®^ assay than from the LD_50_ assay (*p* = 0.02) in the powder formulation; however, a significant difference in variance between the LD_50_ and CBA data sets for the liquid formulation was not observed (*p* = 0.443). In the paired two one-sided *t*-test (TOST) for equivalence, the 90% confidence interval (CI) for the difference between the means for the BoCell^®^ assay and LD_50_ assay was 0.9967–1.044 for powder and 0.9386–1.079 for liquid, which is within the equivalence margin of 0.9–1.1 in both cases. For the powder formulation, Pearson’s correlation coefficient was 0.93, demonstrating a highly positive linear correlation of the potency response over the two product strengths tested and within the acceptance criteria (ρ > 0.80).

**Figure 5:**
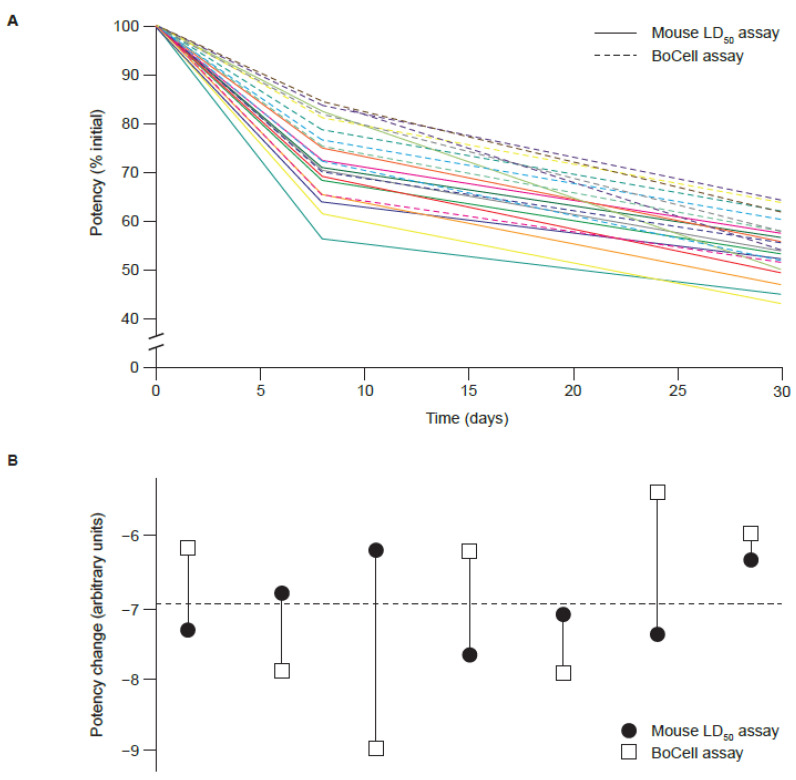
Stability of thermally degraded samples (stored at 40 °C) of aboBoNT-A at 500 U/vial, as detected by the mouse LD_50_ assay and the BoCell^®^ assay. (**A**) Stability profiles of each sample. (**B**) Graphical representation of paired stability slopes. AboBoNT-A, abobotulinumtoxinA; LD_50_, median lethal dose; U, units.

**Figure 6:**
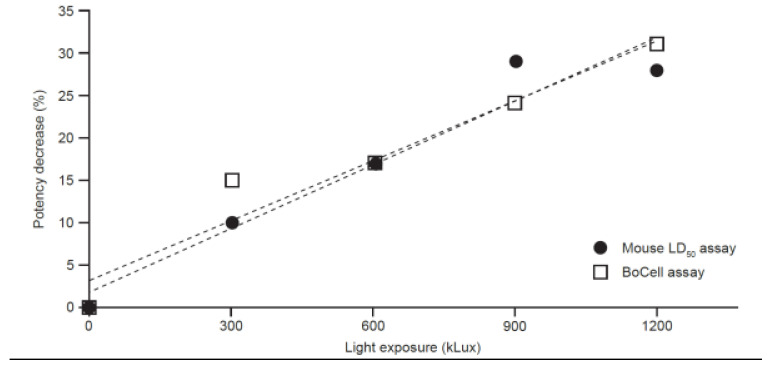
Stability profiles for photodegraded samples (exposed to up to 1200 kLux) of aboBoNT-A at 500 U/vial, as detected by the mouse LD_50_ assay and the BoCell^®^ assay. AboBoNT-A, abobotulinumtoxinA; LD_50_, median lethal dose; U, units.

In the stability analysis, loss in potency over time was determined for each powder formulation batch stored at 40 °C (Figure 5A). Graphical representation of paired stability slope results from the LD_50_ assay and BoCell^®^ assay are shown in Figure 5B. Results from the F-test indicated that the LD_50_ assay slope variance was not significantly different from the variance in the BoCell^®^ assay slope (*p* = 0.06). Potency data from CBA and LD_50_ from the 13 liquid formulation batches stored for 12 months at 2–8 °C are shown in Table 6. When the paired TOST for equivalence was performed, the 90% CI for the difference between the means for the BoCell^®^ assay and LD_50_ assay was 0.8701–1.132 for powder and 0.9072–0.9976 for liquid, which was within the equivalence margin of 0.85–1.15 and 0.90–1.10, respectively. In the photostability study, which was not statistically powered, the BoCell^®^ assay was capable of detecting photodegradation of aboBoNT-A and did so at a similar rate to the mouse LD_50_ assay (Figure 6).

## 3. Discussion

To ensure drug efficacy and patient safety, regulatory authorities worldwide request that manufacturers of BoNTs establish the potency of all batches that are intended for clinical or commercial use.

CBAs have the potential to replace mouse-based LD_50_ assays, leading to a drastic reduction in animal-based testing. However, any assay needs to be rigorously validated, and in cases in which an existing method is already in use, suitable bridging or comparability studies need to be performed before the new assay can be considered acceptable for use by a pharmaceutical company for regulatory batch-testing purposes [11,21,22]. Moreover, the replacement assay must be able to be used in a quality-controlled environment and at the high capacity needed to support commercial production. A cell-based potency assay was developed for onabotulinumtoxinA (Botox, Allergan Inc., Dublin, Ireland) that utilizes differentiated human neuroblastoma SiMa cells and a sandwich enzyme-linked immunosorbent assay readout, which measures BoNT-A-dependent intracellular increase of cleaved SNAP-25 [23]. A CBA has also been developed for incobotulinumtoxinA (Xeomin, Merz, Frankfurt, Germany [24]), although few details about the assay are publicly available.

The data from the BoCell^®^ assay for aboBoNT-A reported here showed selective responsiveness and the ability to reflect key steps of the cellular intoxication process. The assay also showed specificity, linearity, accuracy, repeatability, and intermediate precision, all of which were within the acceptance criteria chosen for aboBoNT-A in this study. The assay was also shown to have suitable performance characteristics for use with both powder and liquid formulations. This is the first example of a CBA being used for potency release of a product containing BoNT in a liquid formulation. These acceptance criteria were established based on the product requirements and the authors’ experience in developing CBAs for biologics, as well as according to requirements from the International Conference of Harmonisation [15] and guidance from the FDA [16]. In this study, the BoCell^®^ assay for aboBoNT-A was equivalent to the mouse-based LD_50_ assay for determining the potency of release samples for both powder and novel ready-to-use liquid formulations in statistically powered comparability assessments. Consistent with the release analysis, the stability analysis demonstrated the closeness of the LD_50_ assay and BoCell^®^ assay data with respect to the mean stability slope observed in the powder formulation batches and the 12-month real-time stability results from the liquid formulation batches. Statistical analysis demonstrated equivalence and a positive linear correlation between the assays.

Based on these findings, the BoCell^®^ assay has the potential to replace the mouse-based LD_50_ assay for potency determination of aboBoNT-A. The mouse LD_50_ assay, which has been the standard for BoNT potency and stability testing, has many serious disadvantages, including the use of many laboratory animals, expensive facilities, the need for highly trained operators, a lack of standardized methodology, and a lack of an international reference standard; all of these result in high variability in the assay and between laboratories [9,11]. The CBA reported here emulates all the essential steps in the BoNT intoxication process and has been shown to be selective, sensitive, specific, robust, and with acceptable levels of reproducibility and precision, which results in low variability in a quality-control setting of BoNT manufacture for patient use. Furthermore, the use of an established cell line facilitates automation of the assay and, in the future, the development and establishment of a common standardized methodology and reference standard for BoNT testing. It is acknowledged that a downside to the use of a genetically engineered immortalized cell line as the basis of an assay method is the potential for instability of the cell line over the lifetime of the assay. To mitigate this risk, the number of division cycles the cell line undergoes after its creation has been minimized by the use of a master cell bank from which working cell banks for the supply of cells to be cultured for the assay are generated. Cell bank stability protocols for both master and working cell banks have also been implemented.

To conclude, this study demonstrates that the BoCell^®^ assay for aboBoNT-A achieves a level of precision and accuracy comparable to the mouse LD_50_ assay and is suitable to be used as a replacement for the LD_50_ assay to support product potency and quality testing of aboBoNT-A. Following the assay validation and comparability studies presented here, this in vitro assay has received approval from regulatory authorities in the European Union (both powder and liquid formulations) and in the USA (FDA; powder formulation) for establishing the potency, for batch release and stability purposes, of aboBoNT-A; submissions and approvals in other geographies are ongoing. In all countries in which Ipsen has received regulatory approval for the use of the CBA methodology presented here, Ipsen is actively using the CBA for aboBoNT-A powder and liquid product batch release and stability testing in place of the mouse LD_50_ method.

## 4. Materials and Methods

### 4.1. BoNT-A Preparations

Research-grade BoNT-A was purchased from Metabiologics Inc. (Madison, WI, USA). HC/A was made at BioSentinel Inc. (Madison, WI, USA), and LC/A was purchased from List Biological Laboratories Inc. (Campbell, CA, USA). AboBoNT-A powder formulation (Dysport^®^) was manufactured by Ipsen (Paris, France); 500 U/vial were used, with further confirmatory analyses being conducted with 125 U/vial for some tests. Vials were reconstituted with complete BoCell^®^ assay media. After reconstitution, vials were pooled to provide a sufficient volume of BoNT-A to perform the assay.

AboBoNT-A liquid formulation (Alluzience^®^) was manufactured by Ipsen (Paris, France); 200 U/vial were used, and vials were pooled before being dialyzed against BoCell^®^ assay media to provide a sufficient volume of aboBoNT-A to perform the assay.

### 4.2. BoCell^®^ Assay Methodology

Cell culture and aboBoNT-A treatment were performed according to BoCell^®^ technology (BioSentinel Inc). Briefly, 20,000 engineered BoCell^®^ Neuro-2a cells/well were exposed to increasing concentrations of aboBoNT-A for a period of 48 h on 96-well microtiter plates. Mean relative fluorescence units (RFU) for YFP and CFP fluorescence output were recorded using a Tecan Infinite M1000Pro (Tecan Group, Männedorf, Switzerland), and mean background fluorescence was determined and subtracted from both emissions spectra. BoNT-A relative potency was calculated with PLA software (Stegmann Systems GmbH, Rodgau, Germany) using the half-maximal effective concentration values generated from fitting either a four-parameter logistic equation (for powder formulation) or a linear regression model (for liquid formulation) to the ratio of blank-subtracted YFP and CFP measurements for nine and seven (powder and liquid formulations, respectively) concentration points for both a reference and test sample. These reference and test curves were tested for parallelism before being fitted using a constrained model in which the values for the slope and the asymptotes were shared between the reference and test sample curves. Reference standards are produced internally by Ipsen Biopharm Ltd. using representative commercial batches of products that have undergone extended product characterization and potency testing.

### 4.3. Validation of the BoCell^®^ Assay to Determine its Capability as a Release Test

The BoCell^®^ assay was validated according to requirements from the International Conference of Harmonisation [19] and guidance from the FDA [20], and based on the authors’ experience in developing CBAs for biologics. A summary of the method validation parameters tested and the acceptance criteria is presented in Table 1.

Specificity was assessed using placebo vials that contained the excipients but no BoNT-A. The ability of the BoCell^®^ assay to measure toxin potency through a mechanism thought to be the mode of action (i.e., cellular binding as its first step and SNARE cleavage activity as the final step) was also tested. BoCell^®^ cells were incubated with either of the following, each in the range of 0.3–100 pM: (a) aboBoNT-A or aboBoNT-A together with HC/A; or (b) BoNT-A or LC/A, which lacks receptor-binding activity. Mean RFU for YFP and CFP fluorescence output were recorded as above.

The accuracy of the BoCell^®^ assay was determined by comparing the potency obtained for each of the 500 U/vial samples at a nominal 50%, 100%, and 130% of the expected concentration. Acceptance criteria for the accuracy study were that recovery for the potency was within 84% and 116% of the target at each of the three levels tested (50%, 100%, and 130% of nominal concentration). Data from the accuracy study were supplemented with assays performed at 70%, 80%, and 120% potency levels to determine the linearity of the method (i.e., its ability to obtain results that are directly proportional to the concentration of analyte in the sample). Estimated potencies were plotted against the expected result for each of the concentrations with an acceptance criterion of a correlation coefficient (*r*^2^) of at least 0.90. For assay repeatability, samples of aboBoNT-A at strengths of 500 U/vial and 125 U/vial were assayed six times, and the precision of the assay was determined by evaluating the %CV obtained for each sample. An acceptance criterion for %CV of 15% or below was used. The intermediate precision of the assay was evaluated using the means and %CVs obtained from 12 assays of 500 U/vial samples, performed by two different operators on different days. Acceptance criteria used were a difference of no more than 10% between operator means and a %CV of 15% or below for different analysts on different days.

### 4.4. Comparability Assessment of Data from the LD_50_ Assay and the BoCell^®^ Assay Using Release and Stability Samples

Statistically powered assessments comparing data from the LD_50_ assay and BoCell^®^ assays were performed for both release and stability samples of aboBoNT-A in the powder formulation at 500 U/vial and in the liquid formulation at 200 U/mL.

In the mouse LD_50_ assay, results were expressed as LD_50_ U/vial, calculated according to the Spearman–Kärber method [25]. Animal experiments were performed by a third-party provider in accordance with the UK Animal (Scientific Procedures) Act 1986, with ethical approval from the UK Home Office granted under license number P8350E94C; 15 April 2019.

In order to demonstrate statistical comparability, an equivalence-based approach was preferred because this allows for an appropriate null hypothesis to be defined and avoids the pitfall of the inappropriate application of a difference test, where statistical equivalence is falsely declared when the test is unable to show a statistical difference between data sets. To analyze the comparability of release samples, a sample size of 30 batches for the powder formulation and at least 10 batches for the liquid formulation was selected. Sample sizes were chosen based on a prospective power calculation with the following assumptions: the ability to identify a difference of 10% between the LD_50_ methods; and BoCell^®^ assay at α of 0.05, with a power of > 90% for powder and > 80% for liquid formulation. Data were analyzed using a paired TOST equivalence test performed in Minitab 17 statistical software (Minitab Ltd., Coventry, UK). In total, 30 powder formulation and 11 liquid formulation batches were tested using the BoCell^®^ assay, and the mean results were expressed as a percentage of data obtained using the LD_50_ assay. A test for the equality of the variances between the LD_50_ and CBA data sets was performed using Bonnett’s method for the powder formulation and Levene’s test for the liquid formulation (the choice of the Levene’s test over the Bonnett’s method was due to the smaller size of the data set for the liquid formulation). A predefined equivalence margin of ±10% was set for the ratio of CBA:LD_50_ comparability data sets. The TOST for equivalence assessed if the difference between the potency determined by both assays was within the equivalence margin by assessing if the 90% CI of the mean of the CBA:LD_50_ ratio data set crossed either the upper or lower bound of the 10% equivalence margin. For the powder data set, a regression analysis was performed by pooling release data from 30 batches of 500 U/vial and seven batches of 300 U/vial data to ensure the regression was performed over a wide enough range of potencies. Pearson’s correlation coefficient was calculated for the relationship between the BoCell^®^ and LD_50_ methods, with an acceptance criterion of ρ > 0.8.

For analyzing the comparability of stability in thermally stressed samples, seven powder formulation batches were placed at 40 ± 2 °C/75 ± 5% relative humidity to obtain sufficient and substantial degradation in a reasonable time frame. Loss of potency over time (slope), according to linear regression, was determined for each batch and data set from each assay. Thirteen liquid formulation batches were placed on stability at 2–8 °C and, after 12 months of storage, were tested for potency in both LD_50_ and CBA methods, with the results being expressed as a percentage of CBA:LD_50_ potency. The test for the equality of the variances and paired equivalence test were performed as above, with predetermined equivalence margins of ±15% for stability slopes for the powder formulation data and ±10% for the difference in CBA:LD_50_ potency at 12 months (2–8 °C) for liquid formulation samples. Non-statistically powered stability data were also generated to determine if there was a difference in the ability of the LD_50_ and BoCell^®^ assays to detect reduced potency due to photostress. Samples from a single batch of aboBoNT-A powder 500 U/vial were exposed to 0, 300, 600, 900, or 1200 kLux.

## Figures and Tables

**Table 1:** Acceptance criteria and results for method validation of the BoCell^®^ assay.

Parameter	Acceptance Criteria		Results for the BoCell^®^ Assay		
	Powder Formulation	Liquid Formulation	Powder Formulation	Liquid Formulation	
Specificity	No response when placebo tested vs. reference standard	No response when placebo tested vs. reference standard	No response seen with placebo	No response seen with placebo	
Linearity	*r*^2^ ≥ 0.90	*r*^2^ ≥ 0.97 CI of the slope for linear regression must include 1 CI for the Y-intercept must include 0	*r*^2^ = 0.98	*r*^2^ = 0.98 CI for slope = 0.99 to 1.13 CI for Y-intercept = −7.51 to 6.45	
Range	Acceptable accuracy, precision, and linearity over the relative potency range of 50–130%	Acceptable accuracy, precision, and linearity over the relative potency range of 50–130%	Accurate, precise, and linear response observed in the range of 50–130%	Accurate, precise, and linear response observed in the range of 50–130%	
Accuracy	Recovery for estimated potency of 84–116% of target potency over the interval of 50–130% relative potency	Recovery for estimated potency of 85–115% of target potency over the interval of 50–130% relative potency	Recovery of 90–103%	Recovery of 106–108%	
Precision–repeatability	%CV ≤ 15%	%CV ≤ 15%	%CV = 3.6% with 500 U/vial %CV = 3.3% with 125 U/vial	%CV = 4%	
Intermediate precision	≤10% difference between operator means	N/A	1.6% difference with 500 U/vial	N/A	
	%CV ≤ 15%	At each of three potency levels (50%, 100%, and 130%), %CV ≤ 15%	%CV = 8.3% with 500 U/vial	Potency level	%CV
				50%	4%
				100%	3%
				130%	5%

CI, confidence interval; %CV, coefficient of variation; N/A, not applicable; U, units.

**Table 2:** Acceptance criteria and results for method validation of the BoCell^®^ assay.

Sample ID	Assay Plate No.	Sample Potency Results Ratio vs. Reference Standard	Unweighted Mean (n = 3)	Individual Assay %CV	Repeatability %CV
500 U/vial	1	0.895	0.913	7.7	3.6
	2	0.853			
	3	0.991			
	1	0.993	0.983	4.4	
	2	1.021			
	3	0.936			
	1	0.850	0.960	10.3	
	2	1.042			
	3	0.987			
	1	1.011	0.994	8.0	
	2	0.906			
	3	1.064			
	1	1.080	0.949	11.9	
	2	0.879			
	3	0.889			
	1	0.975	0.913	6.2	
	2	0.865			
	3	0.898			
125 U/vial	1	0.990	0.981	5.6	3.3
	2	0.923			
	3	1.031			
	1	0.902	0.972	7.1	
	2	1.040			
	3	0.975			
	1	0.949	0.921	2.7	
	2	0.900			
	3	0.914			
	1	1.059	0.903	19.6 ^1^	
	2	0.711			
	3	0.940			
	1	0.989	0.957	6.1	
	2	0.890			
	3	0.993			
	1	0.968	0.970	3.0	
	2	0.941			
	3	1.000			

^1^ The overall repeatability acceptance criteria was a %CV of 15% or below. The assay precision was slightly higher for this replicate but met the requirements of a %CV of 20% or below. %CV, coefficient of variation; ID, identification; U, units.

**Table 3:** Intermediate precision results with 500 U/vial powder formulation samples.

Sample ID	Assay Plate No.	Sample Potency Results Ratio vs. Reference Standard	Unweighted Mean (n = 3)	Individual Assay %CV	Repeatability %CV
Analyst 1	1	0.895	0.913	7.7	10.4
	2	0.853			
	3	0.991			
	1	0.993	0.983	4.4	
	2	1.021			
	3	0.936			
	1	0.850	0.960	10.3	
	2	1.042			
	3	0.987			
	1	1.011	0.994	8.0	
	2	0.906			
	3	1.064			
	1	1.080	0.949	11.9	
	2	0.879			
	3	0.889			
	1	0.975	0.913	6.2	
	2	0.865			
	3	0.898			
	1	0.717	0.714	4.1	
	2	0.683			
	3	0.741			
Analyst 2	1	0.858	0.870	7.1	5.0
	2	0.815			
	3	0.937			
	1	0.959	0.941	1.7	
	2	0.932			
	3	0.932			
	1	0.894	0.843	9.8	
	2	0.748			
	3	0.888			
	1	0.939	0.916	5.7	
	2	0.953			
	3	0.856			
	1	0.953	0.948	3.2	
	2	0.976			
	3	0.916			
**Analyst 1 mean**			0.9180		
**Analyst 2 mean**			0.9036		
**Average**			0.912		
**SD**			0.076		
**%CV**			8.3		

%CV, coefficient of variation; ID, identification; SD, standard deviation; U, units.

**Table 4:** Summary of data for accuracy and intermediate precision assessment from BoCell^®^ assay validation for liquid formulation.

Test Level	Run No.	Weighted Combination Reportable Result (% Rel. Pot.)	Result Expressed as % Recovery	Overall Mean % Recovery (Accuracy)	%CV (Intermediate Precision)
50%	4	51	102	107	4
	5	56	112		
	6	51	102		
	13	54	108		
	14	54	108		
	15	56	112		
100%	1	108		108	3
	2	114			
	3	105			
	10	109			
	11	105			
	12	105			
130%	7	132	102	106	5
	8	143	110		
	9	144	111		
	16	135	104		
	17	145	112		
	18	129	99		

%CV, coefficient of variation. The %CV at all three levels was below 15%. The intermediate precision of the method was considered acceptable at all levels.

**Table 5:** Summary of comparability data for release samples in the BoCell^®^ assay for liquid formulation.

Batch	Purpose of Batch	CNT52120 BAS Batch	CBA, U/mL	LD_50_, U/mL	Ratio, %
L17564	PPQ and stability	L04793	202	201	100
L18325	PPQ and stability	K02858	229	219	105
L19836	PPQ and stability	K02858	201	221	91
L20499	PPQ and stability	L04793	177	209	85
N14521	Potency comparability	L10613	187	224	83
N15416	Potency comparability	L10613	260	232	112
N15791	Potency comparability	L10613	245	214	114
N16407	Potency comparability	L04793	186	190	98
N16906	Potency comparability	L04793	193	216	89
N17823	Potency comparability	L04793	189	172	110
N17580	Potency comparability	L04793	217	178	122
		**Mean**	208	207	101

BAS, bulk active substance; CBA, cell-based assay; LD_50_, median lethal dose; PPQ, process performance qualification; U, units.

**Table 6:** Summary of comparability data for stability samples (12-month storage at 2–8 °C) in the BoCell^®^ assay for liquid formulation.

Batch	Purpose of Batch	CNT52120 BAS Batch	CBA, U/mL	LD_50_, U/mL	Ratio, %
L17564 INV	PPQ and stability	L04793	189	189	100
L18325 INV	PPQ and stability	K02858	192	204	94
L18325 UPR			178	187	95
L19836 INV	PPQ and stability	K02858	174	197	88
L19836 UPR			144	177	81
L20499 INV	PPQ and stability	L04793	187	200	94
L20499 UPR			161	171	94
L13489	Stability	K02858	198	194	102
N15416	Potency comparability	L10613	218	206	106
N15791	Potency comparability	L10613	172	170	101
N16407	Potency comparability	L04793	145	179	81
N16906	Potency comparability	L04793	159	184	86
N17823	Potency comparability	L04793	186	160	116
N17580	Potency comparability	L04793	188	200	94
		**Mean**	178	187	95

BAS, bulk active substance; CBA, cell-based assay; INV, inverted; LD_50_, median lethal dose; PPQ, process performance qualification; U, units; UPR, upright.

## Data Availability

Data underlying this manuscript may be available upon request.
